# Weighted version of Hermite–Hadamard type inequalities for geometrically quasi-convex functions and their applications

**DOI:** 10.1186/s13660-018-1904-7

**Published:** 2018-11-12

**Authors:** Sofian Obeidat, Muhammad Amer Latif

**Affiliations:** Department of Basic Sciences, Deanship of Preparatoray Year Program, University of Hail, Hail, Saudi Arabia

**Keywords:** 26A51, 26D15, 41A55, Hermite–Hadamard inequality, Holder inequality, Convex function, Geometrically symmetric function, Geometrically quasi-convex function, Special means

## Abstract

This paper presents new weighted Hermite–Hadamard type inequalities for a new class of convex functions which are known as geometrically quasi-convex functions. Some applications of these results to special means of positive real numbers have also been presented. These findings have been proved to be useful for researchers working in the fields of numerical analysis and mathematical inequalities.

## Introduction

The past few decades have witnessed an explosion of research on inequalities, including a large number of papers and many fruitful applications. The subject has evoked considerable interest from many mathematicians, and an extensive number of new results have been studied in the literature. It is recognized that in general some specific inequalities provide a useful and essential gadget in the development of various branches of mathematics.

As is notable, the Hermite–Hadamard inequality is one of the most important mathematical inequalities. It states that if $\mu: [ c,d ] \rightarrow \mathbb{R} $ is a convex function, where $c< d$, then

$$\mu \biggl( \frac{c+d}{2} \biggr) \leq\frac{1}{d-c} \int_{c}^{d}\mu ( x ) \,dx\leq\frac{\mu ( c ) +\mu ( d ) }{2}. $$

The importance of the Hermite–Hadamard inequality is due to its role in different branches of modern mathematics such as numerical analysis, functional analysis, and mathematical analysis. It was first observed by Hermite [3] and redeemed later by Hadamard [2]. It is considered as one of the most distinguished results on convex functions due to its strong geometrical significance and applications. Various refinements, generalizations, and applications of the Hermite–Hadamard inequality have appeared in the literature (see for instance [8, 14, 15], and the references therein).

Most recently, Qi and Xi [13] established the Hermite–Hadamard type inequalities for geometrically quasi-convex functions. Inspired and motivated by their work, in this paper, we establish some new weighted Hermite–Hadamard type inequalities using the notion of geometric quasi-convexity, which plays an important role in optimization theory, probability theory, and linear programming. Our inequalities are more general and unique in relation to those given in [13] because of the usage of a weight function, which is assumed to be geometrically symmetric with respect to the geometric mean of the end points of the interval. For further information on quasi-convexity, we refer the reader to [1, 4, 6, 7], and [16]. For further information on Hermite–Hadamard type inequalities for different kinds of convexity assumptions, we refer the interested reader to [5, 6, 9–11], and [1, 12, 14, 15, 17].

Some applications of our results to special means of positive real numbers will also be provided by constructing a geometrically quasi-convex function and a geometrically symmetric function.

Let us recall some important definitions and results before we present the main results of this section.

### Definition 1

([5])

A function $\mu:W\subseteq \mathbb{R} _{+}= ( 0,\infty ) \rightarrow \mathbb{R} $ is said to be geometrically quasi-convex on *W* if

$$\mu \bigl( c^{\theta}d^{1-\theta} \bigr) \leq\sup \bigl\{ \mu ( c ) ,\mu ( d ) \bigr\} $$

for all $c,d\in W$ and $\theta\in [ 0,1 ] $.

### Definition 2

A function $\mu:W\subseteq \mathbb{R} _{+}= ( 0,\infty ) \rightarrow \mathbb{R} $ is said to be geometrically symmetric with respect to $\sqrt{cd}$ if

$$\mu \biggl( \frac{cd}{x} \biggr) =\mu ( x ) $$

for every $x\in W$.

### Lemma 1

*For* $0< c< d$, *we have*

$$\begin{aligned} \Delta_{1} ( c,d ) & := \int_{0}^{1} \bigl\vert \ln \bigl( c^{\frac{1-\theta}{2}}d^{\frac{1+\theta}{2}} \bigr) \bigr\vert \,d\theta \\ & = \textstyle\begin{cases} \frac{-3 ( \ln d ) ^{2}+ ( \ln c ) ^{2}+2 ( \ln c ) ( \ln d ) }{4 ( \ln d-\ln c ) }, & d\leq1, \\ \frac{3 ( \ln d ) ^{2}- ( \ln c ) ^{2}-2 ( \ln c ) ( \ln d ) }{4 ( \ln d-\ln c ) }, & \sqrt {cd}\geq1, \\ \frac{5 ( \ln d ) ^{2}+ ( \ln c ) ^{2}+2 ( \ln c ) ( \ln d ) }{4 ( \ln d-\ln c ) }, & \sqrt {cd}< 1< d, \end{cases}\displaystyle \end{aligned}$$

*and*

$$\begin{aligned} \Delta_{2} ( c,d ) & := \int_{0}^{1}c^{\frac{1-\theta}{2}}d^{\frac{1+\theta}{2}} \bigl\vert \ln \bigl( c^{\frac{1-\theta}{2}}d^{\frac{1+\theta}{2}} \bigr) \bigr\vert \,d\theta \\ & = \textstyle\begin{cases} \frac{2 [ d-\sqrt{cd}+\sqrt{cd}\ln ( \sqrt{cd} ) -d\ln d ] }{\ln d-\ln c}, & d\leq1, \\ \frac{2 [ -d+\sqrt{cd}-\sqrt{cd}\ln ( \sqrt{cd} ) +d\ln d ] }{\ln d-\ln c}, & \sqrt{cd}\geq1, \\ \frac{2 [ 2+\sqrt{cd}\ln ( \sqrt{cd} ) -d-\sqrt{cd}+d\ln d ] }{\ln d-\ln c}, & \sqrt{cd}< 1< d. \end{cases}\displaystyle \end{aligned}$$

### Proof

The proof can be done by simple computations. □

Throughout the manuscript, we will use the following notations for the sake of convenience of the readers:

$$\Omega_{1}(\theta)=c^{\frac{1-\theta}{2}}d^{\frac{1+\theta}{2}}\quad \text{and}\quad \Omega_{2}(\theta)=c^{\frac{1+\theta}{2}}d^{\frac{1-\theta}{2}}. $$

The following result plays a key role in establishing the results of this manuscript.

### Lemma 2

*Let* $\mu:W\subseteq \mathbb{R} _{+}= ( 0,\infty ) \rightarrow \mathbb{R} $ *be a differentiable function on* $W^{\circ}$ *and* *c*, $d\in W^{\circ}$ *with* $c< d$, *and let* $\lambda: [ c,d ] \rightarrow [ 0,\infty ) $ *be a continuous positive mapping and geometrically symmetric to* $\sqrt{cd}$. *If* $\mu^{\prime}\in L [ c,d ] $ *and* $\mu:W\subseteq \mathbb{R} _{+}= ( 0,\infty ) \rightarrow \mathbb{R} $ *is geometrically symmetric with respect to* $\sqrt{cd}$, *then the following equality holds*:

1.1 $$\begin{aligned}& \frac{ ( \ln d ) \mu ( d ) + ( \ln{c} ) \mu(c)}{\ln d+\ln c} \int_{c}^{d}\frac{ ( \ln x ) \lambda(x)}{x}\,dx- \int_{c}^{d}\frac{ ( \ln{x} ) \lambda(x)\mu(x)}{x}\,dx \\& \quad =\frac{ ( \ln d-\ln c ) }{2 ( \ln d+\ln c ) }\biggl[ \int_{0}^{1} \biggl( \int_{\Omega_{2}(\theta)}^{\Omega_{1}(\theta)}\frac{ ( \ln x ) \lambda(x)}{x}\,dx \biggr) \Omega_{1}(\theta )\ln \bigl( {\Omega_{1}(\theta)} \bigr) \mu^{\prime} \bigl( \Omega_{1}(\theta) \bigr) \,d \theta \\& \qquad {} - \int_{0}^{1} \biggl( \int_{\Omega_{2}(\theta)}^{\Omega_{1}(\theta )}\frac{ ( \ln x ) \lambda(x)}{x}\,dx \biggr) \Omega_{2}(\theta )\ln \bigl( \Omega_{2}{(\theta)} \bigr) \mu^{\prime} \bigl( \Omega_{2}(\theta) \bigr) \,d\theta \biggr] . \end{aligned}$$

### Proof

Consider

$$W_{1}=\frac{ ( \ln d-\ln c ) }{2 ( \ln d+\ln c ) }\int_{0}^{1} \biggl( \int_{\Omega_{2}(\theta)}^{\Omega_{1}(\theta)}\frac{ ( \ln x ) \lambda(x)}{x}\,dx \biggr) \Omega_{1}(\theta )\ln \bigl( {\Omega_{1}(\theta)} \bigr) \mu^{\prime} \bigl( \Omega_{1}(\theta) \bigr) \,d \theta $$

which can be written as

$$W_{1}=\frac{1}{\ln d+\ln c} \int_{0}^{1} \biggl( \int_{\Omega_{2}(\theta )}^{\Omega_{1}(\theta)}\frac{ ( \ln x ) \lambda(x)}{x}\,dx \biggr) \ln \bigl( {\Omega_{1}(\theta)} \bigr) \,d\mu\bigl(\Omega_{1}( \theta)\bigr). $$

By integration by parts, making use of the substitution $\Omega _{1}(\theta)=x$ and using the geometric symmetry of *λ* with respect to $\sqrt {cd}$, we have

$$\begin{aligned} W_{1} & =\frac{1}{\ln d+\ln c}\biggl\{ \biggl( \int_{c}^{d}\frac{ ( \ln x ) \lambda(x)}{x}\,dx \biggr) ( \ln{d} ) \mu(d) \\ &\quad {} - \biggl( \int_{\sqrt{cd}}^{\sqrt{cd}}\frac{ ( \ln x ) \lambda(x)}{x}\,dx \biggr) \ln ( \sqrt{cd} ) \mu ( \sqrt {cd} ) \\ &\quad {} -\frac{ ( \ln d-\ln c ) ( \ln d+\ln c ) }{2}\int _{0}^{1}\lambda \bigl(\Omega_{1}(\theta)\bigr)\ln \bigl( {\Omega_{1}(\theta)} \bigr) \mu\bigl(\Omega_{1}(\theta)\bigr)\,d\theta \\ &\quad {} -\frac{ ( \ln d-\ln c ) }{2} \int_{0}^{1} \biggl( \int_{\Omega_{2}(\theta)}^{\Omega_{1}(\theta)}\frac{ ( \ln x ) \lambda(x)}{x}\,dx \biggr) \mu \bigl(\Omega_{1}(\theta)\bigr)\,d\theta\biggr\} \\ &= \frac{ ( \ln{d} ) \mu(d)}{\ln d+\ln c} \int_{c}^{d}\frac{ ( \ln x ) \lambda(x)}{x}\,dx- \frac{ ( \ln d-\ln c ) }{2} \int_{0}^{1}\lambda\bigl(\Omega_{1}( \theta)\bigr)\ln \bigl( {\Omega_{1}(\theta )} \bigr) \mu\bigl( \Omega_{1}(\theta)\bigr)\,d\theta \\ &\quad {} -\frac{ ( \ln d-\ln c ) }{2 ( \ln d+\ln c ) }\int _{0}^{1} \biggl( \int_{\Omega_{2}(\theta)}^{\Omega_{1}(\theta)}\frac{ ( \ln x ) \lambda(x)}{x}\,dx \biggr) \mu \bigl(\Omega_{1}(\theta)\bigr)\,d\theta \\ & =\frac{ ( \ln{d} ) \mu(d)}{\ln d+\ln c} \int_{c}^{d}\frac { ( \ln x ) \lambda(x)}{x}\,dx- \int_{\sqrt{cd}}^{d}\frac{ ( \ln {x} ) \lambda(x)\mu(x)}{x}\,dx \\ &\quad {} -\frac{ ( \ln d-\ln c ) }{2 ( \ln d+\ln c ) }\int _{0}^{1} \biggl( \int_{\Omega_{2}(\theta)}^{\Omega_{1}(\theta)}\frac{ ( \ln x ) \lambda(x)}{x}\,dx \biggr) \mu \bigl(\Omega_{1}(\theta)\bigr)\,d\theta. \end{aligned}$$

Similarly, we can also have

$$\begin{aligned} W_{2}&= \frac{ ( \ln d-\ln c ) }{2 ( \ln d+\ln c ) } \int_{0}^{1} \biggl( \int_{\Omega_{2}(\theta)}^{\Omega_{1}(\theta)}\frac{ ( \ln x ) \lambda(x)}{x}\,dx \biggr) \Omega_{2}(\theta )\ln \bigl( \Omega_{2}{(\theta)} \bigr) \mu^{\prime} \bigl( \Omega_{2}(\theta) \bigr) \,d \theta \\ & =-\frac{ ( \ln{c} ) \mu(c)}{ ( \ln d+\ln c ) }\int_{c}^{d}\frac{ ( \ln x ) \lambda(x)}{x}\,dx+ \int_{c}^{\sqrt {cd}}\frac{ ( \ln{x} ) \lambda(x)\mu(x)}{x}\,dx \\ &\quad {} -\frac{ ( \ln d-\ln c ) }{2 ( ( \ln d+\ln c ) ) } \int_{0}^{1} \biggl( \int_{\Omega_{2}(\theta)}^{\Omega_{1}(\theta )}\frac{ ( \ln x ) \lambda(x)}{x}\,dx \biggr) \mu \bigl(\Omega_{2}(\theta)\bigr)\,d\theta. \end{aligned}$$

Subtracting $W_{2}$ from $W_{1}$, we obtain

$$\begin{aligned} W_{1}-W_{2} & =\frac{ ( \ln d ) \mu ( d ) + ( \ln{c} ) \mu(c)}{\ln d+\ln c} \int_{c}^{d}\frac{ ( \ln x ) \lambda(x)}{x}\,dx- \int_{c}^{d}\frac{ ( \ln{x} ) \lambda(x)\mu (x)}{x}\,dx \\ &\quad {} -\frac{ ( \ln d-\ln c ) }{2 ( \ln d+\ln c ) }\int _{0}^{1} \biggl( \int_{\Omega_{2}(\theta)}^{\Omega_{1}(\theta)}\frac{ ( \ln x ) \lambda(x)}{x}\,dx \biggr) \bigl[ \mu\bigl(\Omega_{1}(\theta )\bigr)-\mu\bigl(\Omega_{2}(\theta) \bigr) \bigr] \,d\theta. \end{aligned}$$

Since *μ* is geometrically symmetric with respect to $\sqrt{cd}$, we have

$$\mu\bigl(\Omega_{1}(\theta)\bigr)=\mu\bigl(\Omega_{2}( \theta)\bigr), $$

and hence

$$\begin{aligned} & \frac{ ( \ln d ) \mu ( d ) + ( \ln{c} ) \mu(c)}{\ln d+\ln c} \int_{c}^{d}\frac{ ( \ln x ) \lambda(x)}{x}\,dx- \int_{c}^{d}\frac{ ( \ln{x} ) \lambda(x)\mu(x)}{x}\,dx \\ &\quad =\frac{ ( \ln d-\ln c ) }{2 ( \ln d+\ln c ) }\biggl[ \int_{0}^{1} \biggl( \int_{\Omega_{2}(\theta)}^{\Omega_{1}(\theta)}\frac{ ( \ln x ) \lambda(x)}{x}\,dx \biggr) \Omega_{1}(\theta )\ln \bigl( {\Omega_{1}(\theta)} \bigr) \mu^{\prime} \bigl( \Omega_{1}(\theta) \bigr) \,d \theta \\ & \qquad {} - \int_{0}^{1} \biggl( \int_{\Omega_{2}(\theta)}^{\Omega _{1}(\theta )}\frac{ ( \ln x ) \lambda(x)}{x}\,dx \biggr) \Omega_{2}(\theta )\ln \bigl( \Omega_{2}{(\theta)} \bigr) \mu^{\prime} \bigl( \Omega_{2}(\theta) \bigr) \,d\theta \biggr] . \end{aligned}$$

 □

## Main results

Based on the above lemmas and definitions, we are able to establish the results of this paper.

### Theorem 1

*Let* $\mu:W\subseteq \mathbb{R} _{+}= ( 0,\infty ) \rightarrow \mathbb{R} $ *be a differentiable function on* $W^{\circ}$ *and* *c*, $d\in W^{\circ}$ *with* $c< d$, *and let* $\lambda: [ c,d ] \rightarrow [ 0,\infty ) $ *be a continuous positive mapping and geometrically symmetric to* $\sqrt{cd}$. *If* $\mu^{\prime}\in L [ c,d ] $, $\mu:W\subseteq \mathbb{R} _{+}= ( 0,\infty ) \rightarrow \mathbb{R} $ *is geometrically symmetric with respect to* $\sqrt{cd}$ *and* $\vert \mu^{{\prime}} \vert $ *is geometrically quasi*-*convex on* $[ c,d ] $, *then*

2.1 $$\begin{aligned}& \biggl\vert \frac{ ( \ln d ) \mu ( d ) + ( \ln {c} ) \mu(c)}{\ln d+\ln c} \int_{c}^{d}\frac{ ( \ln x ) \lambda(x)}{x}\,dx- \int_{c}^{d}\frac{ ( \ln{x} ) \lambda ( x ) \mu(x)}{x}\,dx \biggr\vert \\& \quad \leq\frac{ ( \ln d-\ln c ) ^{2} \Vert \lambda \Vert _{\infty}}{8}\bigl[ \Delta_{2} ( c,d ) \bigl( \sup \bigl\{ \bigl\vert \mu^{\prime} ( \sqrt{cd} ) \bigr\vert , \bigl\vert \mu^{\prime} ( d ) \bigr\vert \bigr\} \bigr) \\& \qquad {} +\Delta_{2} ( d,c ) \bigl( \sup \bigl\{ \bigl\vert \mu^{\prime} ( c ) \bigr\vert , \bigl\vert \mu^{\prime} ( \sqrt{cd} ) \bigr\vert \bigr\} \bigr) \bigr] , \end{aligned}$$

*where* $\Vert \lambda \Vert _{\infty}=\sup_{x\in [ c,d ] } \vert \lambda ( x ) \vert $.

### Proof

By applying the absolute value on both sides of (1.1) and using the properties of the absolute value function, we have

2.2 $$\begin{aligned}& \biggl\vert \frac{ ( \ln d ) \mu ( d ) + ( \ln {c} ) \mu(c)}{\ln d+\ln c} \int_{c}^{d}\frac{ ( \ln x ) \lambda(x)}{x}\,dx- \int_{c}^{d}\frac{ ( \ln{x} ) \lambda(x)\mu (x)}{x}\,dx \biggr\vert \\& \quad \leq\frac{ ( \ln d-\ln c ) \Vert \lambda \Vert _{\infty}}{2 ( \ln d+\ln c ) }\biggl[ \int_{0}^{1} \biggl( \int_{\Omega _{2}(\theta)}^{\Omega_{1}(\theta)}\frac{\ln x}{x}\,dx \biggr) \Omega_{1}(\theta) \bigl\vert \ln \bigl( { \Omega_{1}(\theta)} \bigr) \bigr\vert \bigl\vert \mu^{\prime} \bigl( \Omega_{1}(\theta) \bigr) \bigr\vert \,d\theta \\& \qquad {}+ \int_{0}^{1} \biggl( \int_{\Omega_{2}(\theta)}^{\Omega_{1}(\theta )}\frac{\ln x}{x}\,dx \biggr) \Omega_{2}(\theta) \bigl\vert \ln \bigl( \Omega _{2}{( \theta)} \bigr) \bigr\vert \bigl\vert \mu^{\prime} \bigl( \Omega _{1}(\theta) \bigr) \bigr\vert \,d\theta\biggr] \\& \quad \leq\frac{ ( \ln d-\ln c ) ^{2} \Vert \lambda \Vert _{\infty}}{8}\biggl[ \int_{0}^{1}\Omega_{1}(\theta) \bigl\vert \ln \bigl( {\Omega_{1}(\theta)} \bigr) \bigr\vert \bigl\vert \mu^{\prime} \bigl( \Omega_{1}(\theta) \bigr) \bigr\vert \,d \theta \\& \qquad {}+ \int_{0}^{1}\Omega_{2}(\theta) \bigl\vert \ln \bigl( \Omega _{2}{(\theta)} \bigr) \bigr\vert \bigl\vert \mu^{\prime} \bigl( \Omega _{2}(\theta) \bigr) \bigr\vert \,d\theta\biggr] . \end{aligned}$$

Since $\vert \mu^{\prime} \vert $ is geometrically quasi-convex on $[ c,d ] $, we have

$$\bigl\vert \mu^{\prime} \bigl( \Omega_{1}(\theta) \bigr) \bigr\vert = \bigl\vert \mu^{\prime} \bigl( c^{\frac{1-\theta}{2}}d^{\frac{1+\theta }{2}} \bigr) \bigr\vert \leq\sup \bigl\{ \bigl\vert \mu^{\prime} ( \sqrt{cd} ) \bigr\vert , \bigl\vert \mu^{\prime} ( d ) \bigr\vert \bigr\} $$

and

$$\bigl\vert \mu^{\prime} \bigl( \Omega_{2}(\theta) \bigr) \bigr\vert = \bigl\vert \mu^{\prime} \bigl( c^{\frac{1+\theta}{2}}d^{\frac{1-\theta }{2}} \bigr) \bigr\vert \leq\sup \bigl\{ \bigl\vert \mu^{\prime} ( c ) \bigr\vert , \bigl\vert \mu^{\prime} ( \sqrt{cd} ) \bigr\vert \bigr\} . $$

Hence, by applying Lemma 1 and the above inequalities in (2.1), we have inequality (2.2). Hence the proof of the theorem is accomplished. □

The following interesting result can be deduced from Theorem 1.

### Corollary 1

*If the assumptions of Theorem *1 *are satisfied and if* $\lambda(x)=\frac{1}{ ( \ln x ) ( \ln d-\ln c ) }$ *for all* $x\in [ c,d ] $ *with* $1< c< d<\infty$, *the following inequality holds*:

2.3 $$\begin{aligned}& \biggl\vert \frac{ ( \ln d ) \mu ( d ) + ( \ln {c} ) \mu(c)}{\ln d+\ln c}-\frac{1}{\ln d-\ln c} \int_{c}^{d}\frac {\mu(x)}{x}\,dx \biggr\vert \\& \quad \leq\frac{ ( \ln d-\ln c ) }{8 ( \ln c ) }\bigl[ \Delta_{2} ( c,d ) \bigl( \sup \bigl\{ \bigl\vert \mu^{\prime } ( \sqrt{cd} ) \bigr\vert , \bigl\vert \mu^{\prime} ( d ) \bigr\vert \bigr\} \bigr) \\& \qquad {} +\Delta_{2} ( d,c ) \bigl( \sup \bigl\{ \bigl\vert \mu^{\prime} ( c ) \bigr\vert , \bigl\vert \mu^{\prime} ( \sqrt{cd} ) \bigr\vert \bigr\} \bigr) \bigr] . \end{aligned}$$

A different approach leads us to the following theorem.

### Theorem 2

*Let* $\mu:W\subseteq \mathbb{R} _{+}= ( 0,\infty ) \rightarrow \mathbb{R} $ *be a differentiable function on* $W^{\circ}$ *and* *c*, $d\in W^{\circ}$ *with* $c< d$, *and let* $\lambda: [ c,d ] \rightarrow [ 0,\infty ) $ *be a continuous positive mapping and geometrically symmetric to* $\sqrt{cd}$. *If* $\mu^{{\prime}}\in L [ c,d ] $, $\mu:W\subseteq \mathbb{R} _{+}= ( 0,\infty ) \rightarrow \mathbb{R} $ *is geometrically symmetric with respect to* $\sqrt{cd}$ *and* $\vert \mu^{\prime} \vert ^{\alpha}$ *is geometrically quasi*-*convex on* $[ c,d ] $ *for* $\alpha>1$, *then the following equality holds*:

2.4 $$\begin{aligned}& \biggl\vert \frac{ ( \ln d ) \mu ( d ) + ( \ln {c} ) \mu(c)}{\ln d+\ln c} \int_{c}^{d}\frac{ ( \ln x ) \lambda(x)}{x}\,dx- \int_{c}^{d}\frac{ ( \ln{x} ) \lambda(x)\mu (x)}{x}\,dx \biggr\vert \\& \quad \leq\frac{ ( \ln d-\ln c ) ^{2} \Vert \lambda \Vert _{\infty}}{8} \biggl( \frac{\alpha-1}{\alpha} \biggr) ^{1-\frac{1}{\alpha}} \bigl\{ \bigl[ \Delta_{2} \bigl( c^{\frac{\alpha}{\alpha-1}},d^{\frac {\alpha}{\alpha-1}} \bigr) \bigr] ^{1-\frac{1}{\alpha}} \bigl[ \Delta _{1} ( c,d ) \bigr] ^{\frac{1}{\alpha}} \\& \qquad {}\times \bigl( \sup \bigl\{ \bigl\vert \mu^{\prime} ( \sqrt {cd} ) \bigr\vert , \bigl\vert \mu^{\prime} ( d ) \bigr\vert \bigr\} \bigr) + \bigl[ \Delta_{2} \bigl( d^{\frac{\alpha}{\alpha-1}},c^{\frac{\alpha}{\alpha-1}} \bigr) \bigr] ^{1-\frac{1}{\alpha}} \\& \qquad {}\times \bigl[ \Delta_{1} ( d,c ) \bigr] ^{\frac{1}{\alpha}} \bigl( \sup \bigl\{ \bigl\vert \mu^{\prime} ( c ) \bigr\vert , \bigl\vert \mu^{\prime} ( \sqrt{cd} ) \bigr\vert \bigr\} \bigr) \bigr\} , \end{aligned}$$

*where* $\Vert \lambda \Vert _{\infty}=\sup_{x\in [ c,d ] } \vert \lambda ( x ) \vert $.

### Proof

By applying the absolute value on both sides of (1.1), using the properties of the absolute value function, and by using the Hölder inequality, we have

2.5 $$\begin{aligned}& \biggl\vert \frac{ ( \ln d ) \mu ( d ) + ( \ln {c} ) \mu(c)}{\ln d+\ln c} \int_{c}^{d}\frac{ ( \ln x ) \lambda(x)}{x}\,dx- \int_{c}^{d}\frac{ ( \ln{x} ) \lambda(x)\mu (x)}{x}\,dx \biggr\vert \\& \quad \leq\frac{ ( \ln d-\ln c ) \Vert \lambda \Vert _{\infty}}{2 ( \ln d+\ln c ) }\biggl[ \int_{0}^{1} \biggl( \int_{\Omega _{2}(\theta)}^{\Omega_{1}(\theta)}\frac{\ln x}{x}\,dx \biggr) \Omega_{1}(\theta) \bigl\vert \ln \bigl( { \Omega_{1}(\theta)} \bigr) \bigr\vert \bigl\vert \mu^{\prime} \bigl( \Omega_{1}(\theta) \bigr) \bigr\vert \,d\theta \\& \qquad {}+ \int_{0}^{1} \biggl( \int_{\Omega_{2}(\theta)}^{\Omega_{1}(\theta )}\frac{\ln x}{x}\,dx \biggr) \Omega_{2}(\theta) \bigl\vert \ln \bigl( \Omega _{2}{( \theta)} \bigr) \bigr\vert \bigl\vert \mu^{\prime} \bigl( \Omega _{1}(\theta) \bigr) \bigr\vert \,d\theta\biggr] \\& \quad \leq \frac{ ( \ln d-\ln c ) ^{2} \Vert \lambda \Vert _{\infty}}{8} \biggl[ \biggl( \frac{\alpha-1}{\alpha} \int_{0}^{1}\Omega_{1}^{\frac{\alpha}{\alpha-1}}( \theta) \bigl\vert \ln \bigl( {\Omega_{1}}^{\frac{\alpha}{\alpha-1}}{( \theta)} \bigr) \bigr\vert \,d\theta \biggr) ^{1-\frac{1}{\alpha}} \\& \qquad {}\times\biggl( \int_{0}^{1} \bigl\vert \ln \bigl( {\Omega _{1}(\theta)} \bigr) \bigr\vert \bigl\vert \mu^{\prime} \bigl( \Omega _{1}(\theta) \bigr) \bigr\vert ^{\alpha}\,d\theta \biggr) ^{\frac {1}{\alpha}} \\& \qquad {} + \biggl( \frac{\alpha-1}{\alpha} \int_{0}^{1}\Omega_{2}^{\frac {\alpha }{\alpha-1}}( \theta) \bigl\vert \ln \bigl( \Omega_{2}^{\frac{\alpha }{\alpha-1}}{(\theta)} \bigr) \bigr\vert \,d\theta \biggr) ^{1-\frac{1}{\alpha}} \\& \qquad {}\times \biggl( \int_{0}^{1} \bigl\vert \ln \bigl( { \Omega_{2}(\theta)} \bigr) \bigr\vert \bigl\vert \mu^{\prime} \bigl( \Omega_{2}(\theta) \bigr) \bigr\vert ^{\alpha}\,d\theta \biggr) ^{\frac{1}{\alpha}}\biggr] . \end{aligned}$$

By applying Lemma 1 and the geometric quasi-convexity of $\vert \mu^{{\prime}} \vert ^{\alpha}$ on $[ c,d ] $ for $\alpha>1$, we have

$$\begin{aligned}& \bigl\vert \mu^{\prime} \bigl( \Omega_{1}(\theta) \bigr) \bigr\vert ^{\alpha}\leq\sup \bigl\{ \bigl\vert \mu^{\prime} ( \sqrt{cd} ) \bigr\vert , \bigl\vert \mu^{\prime} ( d ) \bigr\vert \bigr\} , \\& \bigl\vert \mu^{\prime} \bigl( \Omega_{2}(\theta) \bigr) \bigr\vert ^{\alpha}\leq\sup \bigl\{ \bigl\vert \mu^{\prime} ( c ) \bigr\vert , \bigl\vert \mu^{\prime} ( \sqrt{cd} ) \bigr\vert \bigr\} , \\& \int_{0}^{1}\Omega_{1}^{\frac{\alpha}{\alpha-1}}( \theta) \bigl\vert \ln \bigl( \Omega_{1}^{\frac{\alpha}{\alpha-1}}{(\theta)} \bigr) \bigr\vert \,d\theta=\Delta_{2} \bigl( c^{\frac{\alpha}{\alpha-1}},d^{\frac{\alpha}{\alpha-1}} \bigr) , \\& \int_{0}^{1}\Omega_{2}^{\frac{\alpha}{\alpha-1}}( \theta) \bigl\vert \ln \bigl( \Omega_{2}^{\frac{\alpha}{\alpha-1}}{(\theta)} \bigr) \bigr\vert \,d\theta=\Delta_{2} \bigl( d^{\frac{\alpha}{\alpha-1}},c^{\frac{\alpha}{\alpha-1}} \bigr) , \\& \int_{0}^{1} \bigl\vert \ln \bigl( \Omega_{1}{(\theta)} \bigr) \bigr\vert \,d\theta=\Delta_{1} ( c,d ) \quad \text{and}\quad \int_{0}^{1} \bigl\vert \ln \bigl( \Omega_{2}{(\theta)} \bigr) \bigr\vert \,d\theta=\Delta _{1} ( d,c ) . \end{aligned}$$

By using the above facts in (2.5), we get inequality (2.4). This completes the proof of the theorem. □

A consequence of Theorem 2 is the following corollary.

### Corollary 2

*If the conditions of Theorem *2 *are satisfied and if* $\lambda(x)=\frac{1}{ ( \ln x ) ( \ln d-\ln c ) }$ *for all* $x\in [ c,d ] $ *with* $1< c< d<\infty$, *the following inequality holds*:

2.6 $$\begin{aligned}& \biggl\vert \frac{ ( \ln d ) \mu ( d ) + ( \ln {c} ) \mu(c)}{\ln d+\ln c}-\frac{1}{\ln d-\ln c} \int_{c}^{d}\frac {\mu(x)}{x}\,dx \biggr\vert \\& \quad \leq\frac{ ( \ln d-\ln c ) }{8 ( \ln c ) } \biggl( \frac{\alpha-1}{\alpha} \biggr) ^{1-\frac{1}{\alpha}} \bigl\{ \bigl[ \Delta_{2} \bigl( c^{\frac{\alpha}{\alpha-1}},d^{\frac{\alpha}{\alpha-1}} \bigr) \bigr] ^{1-\frac{1}{\alpha}} \bigl[ \Delta_{1} ( c,d ) \bigr] ^{\frac{1}{\alpha}} \\& \qquad {}\times\bigl( \sup \bigl\{ \bigl\vert \mu^{\prime} ( \sqrt {cd} ) \bigr\vert , \bigl\vert \mu^{\prime} ( d ) \bigr\vert \bigr\} \bigr) + \bigl[ \Delta_{2} \bigl( d^{\frac{\alpha}{\alpha-1}},c^{\frac{\alpha}{\alpha-1}} \bigr) \bigr] ^{1-\frac{1}{\alpha}} \\& \qquad {}\times \bigl[ \Delta_{1} ( d,c ) \bigr] ^{\frac{1}{\alpha}} \bigl( \sup \bigl\{ \bigl\vert \mu^{\prime} ( c ) \bigr\vert , \bigl\vert \mu^{\prime} ( \sqrt{cd} ) \bigr\vert \bigr\} \bigr)\bigr\} . \end{aligned}$$

With slightly different assumptions of Theorem 2, one can get the following result.

### Theorem 3

*Let* $\mu:W\subseteq \mathbb{R} _{+}= ( 0,\infty ) \rightarrow \mathbb{R} $ *be a differentiable function on* $W^{\circ}$ *and* *c*, $d\in W^{\circ}$ *with* $c< d$, *and let* $\lambda: [ c,d ] \rightarrow [ 0,\infty ) $ *be a continuous positive mapping and geometrically symmetric to* $\sqrt{cd}$. *If* $\mu^{{\prime}}\in L [ c,d ] $, $\mu:W\subseteq \mathbb{R} _{+}= ( 0,\infty ) \rightarrow \mathbb{R} $ *is geometrically symmetric with respect to* $\sqrt{cd}$ *and* $\vert \mu^{\prime} \vert ^{\alpha}$ *is geometrically quasi*-*convex on* $[c,d ] $ *for* $\alpha>1$ *and* $\alpha>l>0$, *then the following equality holds*:

2.7 $$\begin{aligned}& \biggl\vert \frac{ ( \ln d ) \mu ( d ) + ( \ln {c} ) \mu(c)}{\ln d+\ln c} \int_{c}^{d}\frac{ ( \ln x ) \lambda(x)}{x}\,dx- \int_{c}^{d}\frac{ ( \ln{x} ) \lambda(x)\mu (x)}{x}\,dx \biggr\vert \\& \quad \leq\frac{ ( \ln d-\ln c ) ^{2} \Vert \lambda \Vert _{\infty}}{8} \biggl( \frac{\alpha-1}{\alpha-l} \biggr) ^{1-\frac{1}{\alpha} } \biggl( \frac{1}{l} \biggr) ^{\frac{1}{\alpha}} \bigl\{ \bigl[ \Delta _{2} \bigl( c^{\frac{\alpha-l}{\alpha-1}},d^{\frac{\alpha-l}{\alpha-1}} \bigr) \bigr] ^{1-\frac{1}{\alpha}} \bigl[ \Delta_{2} \bigl( c^{l},d^{l} \bigr) \bigr] ^{\frac{1}{\alpha}} \\& \qquad {}\times\bigl( \sup \bigl\{ \bigl\vert \mu^{\prime} ( \sqrt {cd} ) \bigr\vert , \bigl\vert \mu^{\prime} ( d ) \bigr\vert \bigr\} \bigr) + \bigl[ \Delta_{2} \bigl( d^{\frac{\alpha-l}{\alpha-1}},c^{\frac{\alpha-l}{\alpha-1}} \bigr) \bigr] ^{1-\frac{1}{\alpha }} \\& \qquad {}\times \bigl[ \Delta_{2} \bigl( d^{l},c^{l} \bigr) \bigr] ^{\frac{1}{\alpha}} \bigl( \sup \bigl\{ \bigl\vert \mu^{\prime} ( c ) \bigr\vert , \bigl\vert \mu^{\prime} ( \sqrt{cd} ) \bigr\vert \bigr\} \bigr) \bigr\} , \end{aligned}$$

*where* $\Vert \lambda \Vert _{\infty}=\sup_{x\in [ c,d ] } \vert \lambda ( x ) \vert $.

### Proof

By applying the absolute value on both sides of (1.1), using the properties of the absolute value function and by using the Hölder inequality, we have

2.8 $$\begin{aligned}& \biggl\vert \frac{ ( \ln d ) \mu ( d ) + ( \ln {c} ) \mu(c)}{\ln d+\ln c} \int_{c}^{d}\frac{ ( \ln x ) \lambda(x)}{x}\,dx- \int_{c}^{d}\frac{ ( \ln{x} ) \lambda(x)\mu (x)}{x}\,dx \biggr\vert \\& \quad \leq\frac{ ( \ln d-\ln c ) \Vert \lambda \Vert _{\infty}}{2 ( \ln d+\ln c ) }\biggl[ \int_{0}^{1} \biggl( \int_{\Omega _{2}(\theta)}^{\Omega_{1}(\theta)}\frac{\ln x}{x}\,dx \biggr) \Omega_{1}(\theta) \bigl\vert \ln \bigl( { \Omega_{1}(\theta)} \bigr) \bigr\vert \bigl\vert \mu^{\prime} \bigl( \Omega_{1}(\theta) \bigr) \bigr\vert \,d\theta \\& \qquad {} + \int_{0}^{1} \biggl( \int_{\Omega_{2}(\theta)}^{\Omega_{1}(\theta )}\frac{\ln x}{x}\,dx \biggr) \Omega_{2}(\theta) \bigl\vert \ln \bigl( \Omega _{2}{( \theta)} \bigr) \bigr\vert \bigl\vert \mu^{\prime} \bigl( \Omega _{1}(\theta) \bigr) \bigr\vert \,d\theta\biggr] \\ & \quad \leq\frac{ ( \ln d-\ln c ) ^{2} \Vert \lambda \Vert _{\infty}}{8}\biggl[ \biggl( \frac{\alpha-1}{\alpha-l} \int_{0}^{1}\Omega _{1}^{\frac{\alpha-l}{\alpha-1}}( \theta) \bigl\vert \ln \bigl( {\Omega_{1} }^{\frac{\alpha-l}{\alpha-1}}{(\theta)} \bigr) \bigr\vert \,d\theta \biggr) ^{1-\frac{1}{\alpha}} \\ & \qquad {}\times \biggl( \frac{1}{l} \int_{0}^{1} \bigl\vert \ln \bigl( { \Omega_{1}}^{l}{(\theta)} \bigr) \bigr\vert \Omega_{1}^{l}(\theta) \bigl\vert \mu^{\prime} \bigl( \Omega_{1}(\theta) \bigr) \bigr\vert ^{\alpha}\,d \theta \biggr) ^{\frac{1}{\alpha}} \\ & \qquad {}+ \biggl( \frac{\alpha-1}{\alpha-l} \int_{0}^{1}\Omega_{2}^{\frac{\alpha -l}{\alpha-1}}( \theta) \bigl\vert \ln \bigl( \Omega_{2}^{\frac{\alpha-l}{\alpha-1}}{(\theta)} \bigr) \bigr\vert \,d\theta \biggr) ^{1-\frac {1}{\alpha}} \\ & \qquad {}\times \biggl( \frac{1}{l} \int_{0}^{1} \bigl\vert \ln \bigl( {\Omega _{2}}^{l}{(\theta)} \bigr) \bigr\vert \Omega_{1}^{l}(\theta) \bigl\vert \mu^{\prime} \bigl( \Omega_{2}(\theta) \bigr) \bigr\vert ^{\alpha}\,d \theta \biggr) ^{\frac{1}{\alpha}}\biggr] . \end{aligned}$$

By using similar arguments as in proving (2.4), we get (2.8). □

As a natural consequence of Theorem 3, we get the following result.

### Corollary 3

*If the hypothesis of Theorem *3 *is satisfied and if* $\lambda(x)=\frac{1}{ ( \ln x ) ( \ln d-\ln c ) }$ *for all* $x\in [ c,d ] $ *with* $1< c< d<\infty$, *the following inequality holds*:

2.9 $$\begin{aligned}& \biggl\vert \frac{ ( \ln d ) \mu ( d ) + ( \ln {c} ) \mu(c)}{\ln d+\ln c}-\frac{1}{\ln d-\ln c} \int_{c}^{d}\frac {\mu(x)}{x}\,dx \biggr\vert \\ & \quad \leq\frac{ ( \ln d-\ln c ) }{8 ( \ln c ) } \biggl( \frac{\alpha-1}{\alpha-l} \biggr) ^{1-\frac{1}{\alpha}} \biggl( \frac{1}{l} \biggr) ^{\frac{1}{\alpha}}\bigl\{ \bigl[ \Delta_{2} \bigl( c^{\frac{\alpha-l}{\alpha-1}},d^{\frac{\alpha-l}{\alpha-1}} \bigr) \bigr] ^{1-\frac{1}{\alpha}} \bigl[ \Delta_{2} \bigl( c^{l},d^{l} \bigr) \bigr] ^{\frac{1}{\alpha}} \\ & \qquad {}\times \bigl( \sup \bigl\{ \bigl\vert \mu^{\prime} ( \sqrt {cd} ) \bigr\vert , \bigl\vert \mu^{\prime} ( d ) \bigr\vert \bigr\} \bigr) + \bigl[ \Delta_{2} \bigl( d^{\frac{\alpha-l}{\alpha-1}},c^{\frac{\alpha-l}{\alpha-1}} \bigr) \bigr] ^{1-\frac{1}{\alpha }} \\ & \qquad {}\times \bigl[ \Delta_{2} \bigl( d^{l},c^{l} \bigr) \bigr] ^{\frac{1}{\alpha}} \bigl( \sup \bigl\{ \bigl\vert \mu^{\prime} ( c ) \bigr\vert , \bigl\vert \mu^{\prime} ( \sqrt{cd} ) \bigr\vert \bigr\} \bigr)\bigr\} . \end{aligned}$$

## Applications to special means

In this section, we show how the above established inequalities of Hermite–Hadamard type can be used to obtain the inequalities for special means.

For positive numbers $c>0$ and $d>0$ with $c\neq d$,

$$A ( c,d ) =\frac{c+d}{2}, \qquad L ( c,d ) =\frac {d-c}{\ln d-\ln c}, \qquad G ( c,d ) =\sqrt{cd}$$

and

$$L_{p} ( c,d ) = \textstyle\begin{cases} [ \frac{d^{p+1}-c^{p+1}}{ ( p+1 ) ( d-c ) } ] ^{\frac{1}{p}}, & p\neq-1,0, \\ L ( c,d ) , & p=-1, \\ \frac{1}{e} ( \frac{d^{d}}{c^{c}} ) ^{\frac{1}{d-c}}, & p=0 \end{cases} $$

are the arithmetic mean, the logarithmic mean, the geometric mean, and the generalized logarithmic mean of order $p\in \mathbb{R} $, respectively. For further information on means, we refer the readers to [12] and [17], and the references therein.

Let us consider $\mu: ( 0,\infty ) \rightarrow \mathbb{R} $ defined by

$$\mu ( x ) =x+\frac{cd}{x}. $$

It is clear that the function *μ* is geometrically symmetric about $\sqrt{cd}$.

We observe that

$$\mu^{{\prime}} ( x ) =1-\frac{cd}{x^{2}}. $$

Then

$$\bigl\vert \mu^{\prime} ( x ) \bigr\vert = \textstyle\begin{cases} \frac{cd}{x^{2}}-1, & c\leq x\leq\sqrt{cd}, \\ 1-\frac{cd}{x^{2}}, & \sqrt{cd}\leq x\leq d. \end{cases} $$

Clearly

$$\bigl\vert \mu^{\prime} ( c ) \bigr\vert > \bigl\vert \mu ^{\prime } ( d ) \bigr\vert . $$

Thus

$$\sup \bigl\{ \bigl\vert \mu^{\prime} ( c ) \bigr\vert , \bigl\vert \mu^{\prime} ( d ) \bigr\vert \bigr\} =\frac{d}{c}-1. $$

Note that

$$\bigl\vert \mu^{\prime} \bigl( c^{1-\theta}d^{\theta} \bigr) \bigr\vert = \textstyle\begin{cases} ( \frac{d}{c} ) ^{1-2\theta}-1, & \theta\leq\frac{1}{2}, \\ ( \frac{d}{c} ) ^{2\theta-1}-1, & \theta\geq\frac{1}{2}. \end{cases} $$

If $\theta\leq\frac{1}{2}$

$$\bigl\vert \mu^{\prime} \bigl( c^{1-\theta}d^{\theta} \bigr) \bigr\vert \leq\frac{d}{c}-1= \bigl\vert \mu^{\prime} ( c ) \bigr\vert . $$

Thus

$$\bigl\vert \mu^{\prime} \bigl( c^{1-\theta}d^{\theta} \bigr) \bigr\vert \leq\sup \bigl\{ \bigl\vert \mu^{\prime} ( c ) \bigr\vert , \bigl\vert \mu^{\prime} ( d ) \bigr\vert \bigr\} \quad \text{for }\theta \leq\frac{1}{2}. $$

If $\theta\geq\frac{1}{2}$, then

$$\bigl\vert \mu^{\prime} \bigl( c^{1-\theta}d^{\theta} \bigr) \bigr\vert = \biggl( \frac{d}{c} \biggr) ^{2\theta-1}-1. $$

Note that $\frac{c}{d}<1$ implies

$$\frac{d}{c}+ \biggl( \frac{d}{c} \biggr) ^{2\theta-1}> \frac{d}{c}+\frac{c}{d}>2, $$

which implies that

$$\bigl\vert \mu^{\prime} ( c ) \bigr\vert > \bigl\vert \mu ^{\prime } \bigl( c^{1-\theta}d^{\theta} \bigr) \bigr\vert . $$

Therefore, we have

$$\sup \bigl\{ \bigl\vert \mu^{\prime} ( c ) \bigr\vert , \bigl\vert \mu^{\prime} ( d ) \bigr\vert \bigr\} \geq \bigl\vert \mu ^{\prime} \bigl( c^{1-\theta}d^{\theta} \bigr) \bigr\vert \quad \text{for }\theta\geq\frac{1}{2}. $$

Hence it is proved that $\vert \mu^{\prime} ( x ) \vert $ is geometrically quasi-convex.

### Proposition 1

*If* $0< c< d<\infty$, *then the following inequality of means holds true*:

3.1 $$\begin{aligned}& \biggl\vert \frac{A ( ( \ln c ) \mu ( c ) , ( \ln d ) \mu ( d ) ) }{A ( \ln c,\ln d ) }-2A ( c,d ) \biggr\vert \\& \quad \leq\frac{ ( \ln d-\ln c ) }{8 ( \ln c ) } \bigl[ d\Delta_{2} ( c,d ) +c \Delta_{2} ( d,c ) \bigr] . \end{aligned}$$

### Proof

The function $\mu: ( 0,\infty ) \rightarrow \mathbb{R} $ defined by

$$\mu ( x ) =x+\frac{cd}{x} $$

is geometrically symmetric about $\sqrt{cd}$ and $\vert \mu^{\prime} ( x ) \vert $ is geometrically quasi-convex. By considering the above defined function for Corollary 1, we get inequality (3.1). □

### Proposition 2

*If* $0< c< d<\infty$, *then the following inequality of means holds true*:

3.2 $$\begin{aligned}& \bigl\vert 2A \bigl( ( \ln c ) \mu ( c ) , ( \ln d ) \mu ( d ) \bigr) \bigl[ G ( c,d ) -c \bigr] \\& \qquad {} +cA ( c\ln c,d\ln d ) +cA ( c,d ) \ln d+G ( c,d ) \ln d^{2} \bigr\vert \\& \quad \leq\frac{G ( c,d ) ( \ln d-\ln c ) ^{2}}{8} \bigl[ d\Delta_{2} ( c,d ) +c \Delta_{2} ( d,c ) \bigr] . \end{aligned}$$

### Proof

The function $\mu: ( 0,\infty ) \rightarrow \mathbb{R} $ defined by

$$\mu ( x ) =x+\frac{cd}{x} $$

is geometrically symmetric about $\sqrt{cd}$ and $\vert \mu^{\prime } ( x ) \vert $ is geometrically quasi-convex. The function $\lambda: [ c,d ] \rightarrow [ 0,\infty ) $ defined by

$$\lambda ( x ) = \textstyle\begin{cases} \frac{cd}{x}, & x\geq\sqrt{cd}, \\ x, & x\leq\sqrt{cd}\end{cases} $$

is geometrically symmetric about $\sqrt{cd}$. By considering the above defined functions for Theorem 1, we get inequality (3.1). □

### Remark 1

A number of interesting inequalities can be obtained from the other results of this manuscript if we use the functions defined in Proposition 1 and Proposition 2.

## Conclusion

In this paper, a new weighted identity, which involves a continuous positive mapping geometrically symmetric about $\sqrt{cd}$ and a differentiable mapping also geometrically symmetric about $\sqrt{cd}$, is presented. Some new inequalities are developed by applying mathematical analysis and the geometric symmetry of a continuous positive mapping and a differentiable mapping whose derivatives in absolute value are geometrically quasi-convex. Some applications of the proved results are given for special means of positive real numbers. The results of this paper may stimulate further research for the researchers working in this filed.
